# Cross-comparison of microbiota in the oropharynx, hypopharyngeal squamous cell carcinoma and their adjacent tissues through quantitative microbiome profiling

**DOI:** 10.1080/20002297.2022.2073860

**Published:** 2022-05-10

**Authors:** Hui-Ching Lau, Yujie Shen, Huiying Huang, Xiaohui Yuan, Mengyou Ji, Hongli Gong, Chi-Yao Hsueh, Liang Zhou

**Affiliations:** a Department of Otorhinolaryngology, Eye & ENT Hospital, Fudan University, Shanghai, PR, China; b Shanghai Key Clinical Disciplines of Otorhinolaryngology, Shanghai, PR, China

**Keywords:** Quantitative microbiome profiling, HPSCC, QIIME2, ASV, cross-sectional study

## Abstract

**Aims:**

To clarify the absolute abundance of microbial communities on hypopharyngeal squamous cell carcinoma and their correlation to those in the oropharynx.

**Methods:**

Clinical data, swabs, and tissue samples from 27 HPSCC patients were collected in this study and divided into three sampling groups: 19 oropharyngeal mucosa (OPM), 27 hypopharyngeal carcinomas tissues (HC), and 26 corresponding adjacent tissues (AT). Relative microbiome profiling (RMP), and quantitative microbiome profiling (QMP) of 16S rRNA amplicon sequencing were used for analysis.

**Results:**

Beta-diversity showed that abundance and phylogenetic tree in OPM group were less when compared to either HC and AT. Although HC and AT were found to have similar microbiota, Bray-Curtis based beta-diversity still highlighted differences. *Fusobacterium, Porphyromonas, Haemophilus*, and *Peptostreptococcus* at the genus level in OPM were positively correlated with HC. After categorizing HC through TNM staging, the abundance of genera *Fusobacterium, Parvimonas*, and *Dialister* were found to be enhanced in higher T classifications (T3-4) and advanced stages (Ⅳ).

**Conclusions:**

QMP yielded more comprehensive results than RMP. Dysbiosis was found in OPM groups and could be used to narrow down differential microbiome for the HC group. Genera of *Parvimonas, Fusobacterium*, and *Dialister* were deemed asrisk factors of advanced HPSCC.

## Introduction

Hypopharyngeal squamous cell carcinoma (HPSCC) accounts for approximately 3% of head and neck carcinoma (HNC) and often has a worse prognosis compared to other HNCs. Initial diagnoses for patients with HPSCC are often late. Even though different treatment strategies are available, the five-year overall survival rate has remained at approximately 30–35% for advanced HPSCC [1]. To date, potential biomarkers are still being pursued, with microorganisms being a favorable contender as they are responsible for approximately 13% of human carcinoma [2]. The dynamics of various environments, ranging from the organic bodies to the sea or soil, could be shaped by the complex microbial communities. Dysbiosis and variation in microbial communities have been proven to be associated with the formation and progression of carcinoma. Some bacteria at a species level such as *Fusobacterium nucleatum, Escherichia coli*, and *Bacteroides fragilis* could induce DNA damage, suppress the immune function, and create a proinflammatory environment, resulting in the formation and progression of colorectal carcinoma (CRC), where treatment using antibacterial drugs could benefit the turnover to different degrees [3].

Testing for relative microbial load and their connection to bodies and environment has become more accessible and accurate thanks to relative microbiome profiling (RMP) methods, such as high-throughput technology Illumina MiSeq/HiSeq platform on 16S ribosomal RNA (16S rRNA) amplicon and whole-genome sequencing (WGS) [4]. However, repetitive sequence detection in a single genome amplifies cross-organismal repeats through WGS could lead to uneven results, translating to high operational costs, and large information dumps [5]. 16S rRNA amplicon profiling, on the other hand, has disadvantages in lack of specificity and primer bias [6]. Therefore, simply relying on relative microbiome profiling (RMP) methods is not persuasive enough to connect the variation of biodiversity with their biological specialties, especially when taking into account treatments received and disease state [7]. To address this issue and to obtain reliable large-scale data, Quantitative Microbiome Profiling (QMP) was introduced to complement RMP [8]. QMP provides interpretative information based on the amount of microbiome sequencing data. Traditional methods such as flow cytometry, fluorescence microscopy, and qPCR are feasible approaches to measure the absolute microbial abundances [9,10]. Lin et al. introduced another way to estimate the abundance of microorganisms by adding spike-in known amounts of internal DNA standards to explore the rapidly expanding and changing bacteria in the ocean [11]. To our understanding, the use of spike-in to assess the absolute abundance of microbiota in an HNC study is novel.

In the past five years, the variations in the oral microbiome were shown to have the potential in acting as prognosis predictors in HNC, especially for oral squamous cell carcinoma (OSCC) [12], tonsil carcinoma [13] and laryngeal carcinoma [14]. The application of differential microbiome was also found in several longitudinal therapeutic studies. Here, we conducted a cross-sectional study by collecting up to three distinct samples from each patient with HPSCC: the tissue of the hypopharyngeal carcinoma (HC), their adjacent tissue (AT), and swab sampling on oropharyngeal mucosa (OPM). We wished to answer whether microbiota changes in OPM can be considered as changes in flora structure on the tumor site and whether combined QMP and RMP could benefit in interpreting the variation in these three groups.

## Materials and methods

### Subject recruitment, data collection

A total of 32 HPSCC patients from December 2017 to December 2019 at Eye and ENT Hospital, Fudan University, Shanghai were included. The 8^th^ edition AJCC TNM staging was used to assess clinical data. The TNM staging, containing primary tumor site (T classification), regional lymph node involvement (N classification), and the presence of distant metastatic spread (M classification), could help clinicians assess the prognosis of carcinoma [15]. The inclusion criteria in the HPSCC group were as follows: (1) biopsy confirmed pathologic hypopharyngeal squamous cell carcinoma; (2) completed clinical and laboratory data in whole administration; (3) patients must have accepted at least two samplings from different locations. The exclusion criteria were as follows, and the number of cases excluded are attached as (n): (1) accompanied by other tumors in five years (n = 1). (2) Cessation of antibiotic intake for less than three months [16]: three patients that recieved antibiotics within two weeks of sampling were excluded (n = 3). One obtained acute upper respiratory tract infection after induced chemotherapy and two others received quadruple therapy for Helicobacter pylori-associated chronic gastritis. (3) unable to pass primary quality detection (n = 1). A total of 27 patients with HPSCC were enrolled and the locations from which samples were obtained are listed in **Supplementary Table 1**. Written informed consent by all participants was obtained. Ethical approval was granted by the Ethical Committees of Eye and ENT Hospital, Fudan University.

### Sample collection, DNA extraction

Based on the National Institutes of Health Human Microbiome Project (http://hmpdacc.org/doc/HMP_Clinical_Protocol.pdf), two swabs (FLOQ Swabs, COPAN) were used to sample microbiota on the oropharyngeal mucosa (OPM). Patients were instructed not to perform oral hygiene practices before sample grabbing. The time of grabbing was set at least two hours before surgical treatment. A total of 20 mg of scratched carcinoma tissue and their adjacent tissues were sterilely collected. The adjacent tissues (AT) were obtained at least 2 cm distance away from the edge of the carcinoma. The swabs and other samples were then quickly stored at −80°C for further DNA extraction. Total DNA extraction was extracted by following the instruction of the FastDNA® SPIN Kit for Soil (MP Biomedicals, Santa Ana, CA). The Qubit 3.0 spectrophotometer was used to detect the quantity of genome DNA, and Agilent Bioanalyzer (ABI) 3730 system was used to assess the sequence in the library that was between 120–200 bp without nonspecific amplification. Profiling was set to **10 ng/ul** and total content was required to be **≥500 ng**.

### Preparing the absolute and relative 16S rRNA amplicon sequencing

Accu16S^TM^ system was performed to define absolute quantification of 16S rRNA sequencing (QMP). The different spike-ins with identical conserved regions could recognize natural 16S rRNA genes and variable regions through replaced random sequences with approximately 40% GC content, which were artificially designed and synthesized. Then, an appropriate proportion of spike-ins mixture with known gradient copy numbers was added to the sample DNA. The V3-V4 hypervariable regions of the 16S rRNA gene and spike-ins were amplified with the primers 341 F 5'-CCTACGGGNGGCWGCAG-3' and 805 R 5'-GACTACHVGGGTATCTAATCC-3' with 250 bp paired-end reads through using Illumina NovaSeq 6000 sequencer. The relative amplicon sequencing of RMP adopted the same process without carrying out the spike-ins in samples. All samples that went through RMP had the lowest number of tags set as standard, thereafter standardized to the same amount with proportional ASVs.

### Illumina read data processing and analysis

The raw read sequences were processed in QIIME2. The adaptor and primer sequences were trimmed using the ‘cutadapt’ plugin, that is, **Primer F** = Illumina adapter sequence 1+ CCTACGGGNGGCWGCAG; **Primer R** = Illumina adapter sequence 2+ GACTACHVGGGTATCTAATCC; **Illumina adapter sequence 1** = AATGATACGGCGACCACCGAGATCTACACXXXXXXXXTCGTCGGCAGCGTCAGATGTGTATAAGAGACAG; **Illumina adapter sequence 2** = CTGTCTCTTATACACATCTCCGAGCCCACGAGACXXXXXXXXATCTCGTATGCCGTCTTCTGCTTG. DADA2 plugin was performed to validate the quality and to identify amplicon sequence variants (ASVs). Taxonomic assignments of ASV representative sequences were performed by Naive Bayes classifier, which was trained on the RDP (version 11.5) with a confidence threshold of 0.8. The spike-in sequences were identified and reads were calculated. The standard curves for each sample were generated for calculating the read counts and the absolute copy number of each ASV in each sample. The unit of measurement was set as ASV copies/ng DNA. The spike-in sequence would be further depleted in the following analysis.

### Data analysis

QIIME 2.0 software was used to calculate both alpha- and beta-diversity, and R software was used to visualize the results. In alpha diversity, the observed species index and the ACE index were exploited to assess the richness of the ASVs community. Shannon index was used to assess the evenness of community diversity. Bray-Curtis and Weighted UniFrac analyses were implemented to identify the existence and phylogenetic relationship of beta-diversity. Principal coordinate analysis (PCoA) based on Weighted UniFrac distance matrices and Bray-Curtis were performed to obtain principal coordinates and visualize complex multidimensional data. Nonmetric multidimensional scaling (NMDS) provided a non-linear structural model of biological ecology. Adonis analysis was used to compare differential microbial communities between groups. To identify different genera of bacterial composition between groups, the LEfSe method was employed. LEfSe (LDA Effect Size) was used to explore the high dimensional bacterial taxon between groups with the evolutionary tree in the cladogram. Linear discriminant analysis (LDA) over 2.0 was deemed as high-dimension and p < 0.05 as statistically meaningful. Wilcoxon rank-sum test was used to validate the absolute abundance of microbiota in different groups, which were categorized by T classification and TNM staging. The second version Phylogenetic investigation of communities by reconstruction of unobserved states (PICRUSt2) was a bioinformatic tool used to predict the functional potential of marker gene sequence profile in a bacterial community, which contains a larger database of reference genes and shows higher accuracy and flexibility than PICRUSt. Ribosomal RNA operons (rrn) database (ver.5.6, https://rrndb.umms.med.umich.edu) was used to deduct the closed reference metagenome of each 16S rRNA sequence (ASVs). PICRUSt2 tool was used to explore the molecular functions based on KEGG pathways [17].

### Nucleotide sequence accession number

The datasets of the raw sequence files and metagenome used in this study were uploaded and deposited in Genome Sequence Archive (GSA) in China National Center for Bioinformation (CNCB) with the accession number **CRA004979** [18,19].

## Results

### The demographic of enrolled HPSCC patients and their microbiota diversity

The demographics of different clinical characteristics in 27 HPSCC patients were detailed in Table 1. The total count of the demultiplexed sequence was 9,759,230 (median: 153,518.0). A total of 5404 ASVs in QMP were included, averaging 185 phyla, 179 classes, 178 orders, 176 families, and 172 genera annotated in the QIIME2 database. The standard curve based on a linear equation was testified for each sample. In RMP, 2816 ASVs were detected, covering 126 phyla, 123 classes, 122 orders, 121 families, and 118 genera on average.

**Table 1.** Clinical characteristics of 27 enrolled HPSCC patients

Characteristics	Number of cases (proportion)
Age (years)	59.51 ± 7.80
HTN (no/ yes)	15(55.6%)/ 12(44.4%)
DM (no/ yes)	26(96.3%)/ 1(3.7%)
Smoking history (no/ yes)	5(18.5%)/ 22(81.5%)
Drinking history (no/ yes)	6(22.2%)/ 21(77.8%)
Induced chemotherapy before surgery (no/ yes)	21(77.8%)/ 6(22.2%)
Pathological types (PS/ PC/ PP)	23(85.2%)/ 2(7.4%)/ 2(7.4%)
Surgery options (PLPH/ TLPH/ TLTH)	8(29.6%)/ 13(48.1%)/ 6(22.2%)
cT classification (T2/ T3/ T4)	5(18.5%)/ 15(55.6%)/ 7(25.9%)
cN classification (N0/ N+)	7(25.9%)/ 20(74.1%)
pT classification (T2/ T3/ T4)	8(29.6%)/ 15(55.6%)/ 4(14.8%)
pN classification (N0/ N+)	9(33.3%)/ 18(66.7%)
TNM stage (I-II/III-IV)	4(14.8%)/ 23(85.2%)
ENE (no/ yes)	20(74.1)/ 7(25.9)
Tumor diameters (≤ 4 cm/ > 4 cm)	17(63.0%)/ 10(37.0%)
Size of lymph node (≤ 3 cm/ > 3 cm)	19(70.4%)/ 8(29.6%)

Abbreviation: HTN, hypertension; DM, diabetes mellitus; ENE, extranodal extension; cT, clinical judgment tumor classification; cN, clinical judgment node metastasis classification; pT, pathological judgment tumor classification; pN, pathological judgment node metastasis classification; PS, Pyriform sinus; PC, post-cricoid region; PP, posterior pharyngeal wall; PLPP, partial laryngectomy and partial pharyngectomy; TLPH, total laryngectomy and partial pharyngectomy; TLTH, total laryngectomy and total pharyngectomy

### The overall variation of microbial communities between QMP and RMP

**Figure 1.**
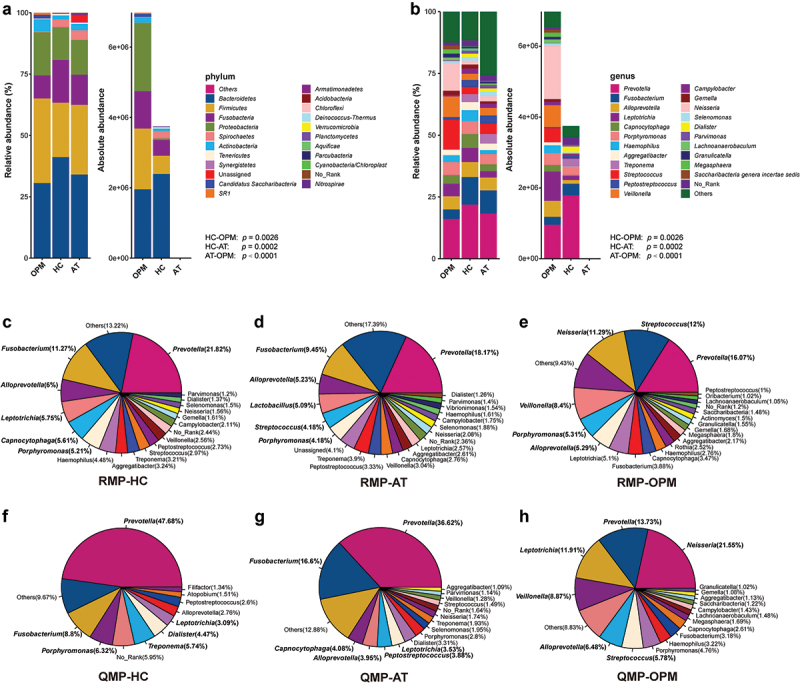
**The relative and absolute composition of OPM, HC, and AT in barplot and pie plot**. (A) phylum level in barplot. (B) genus level in barplot. The amount of ASVs in these three groups presents a significant difference under Kruskal-Wallis test (HC-AT: *p* = 0.0002; HC-OPM: *p* = 0.0026; AT-OPM: *p* < 0.0001). Pie plot depicted the different proportions of genera in RMP (C-E) and QMP (F-H).

Different compositions of microbiota in QMP and RMP were found at the level of phylum and genera. The total quantity of ASVs was deemed as the amount of genome DNA of microbial communities. The amount of microbiome in the OPM group was much higher than in HC via QMP (Kruskal–Wallis test: *p* = 0.0048). The microbiome in AT groups had the lowest amount at the basis of phylum and genus when compared to HC and OPM (AT-OPM: *p* = 0.0001; AT-OPM: *p* < 0.0001), (Figure 1A-B).

We discovered that pie plots, composed of genera-based microbiota in the categorized group were also varied between RMP and QMP (Figure 1C-H). The top six identified absolute genera of microbiota in AT group were *Prevotella, Fusobacterium, Capnocytophaga, Alloprevotella, Peptostreptococcus*, and *Leptotrichia*. The top six identified absolute genera in HC group were *Prevotella, Fusobacterium, Porphyromonas, Treponema, Dialister*, and *Leptotrichia*. The top six identified absolute genera in the OPM group were *Neisseria, Prevotella, Leptotrichia, Veillonella, Alloprevotella*, and *Streptococcus*. The variation of microbiota through QMP or RMP could be seen in the line graph (**Supplementary Figure 1**).

**Figure 2.**
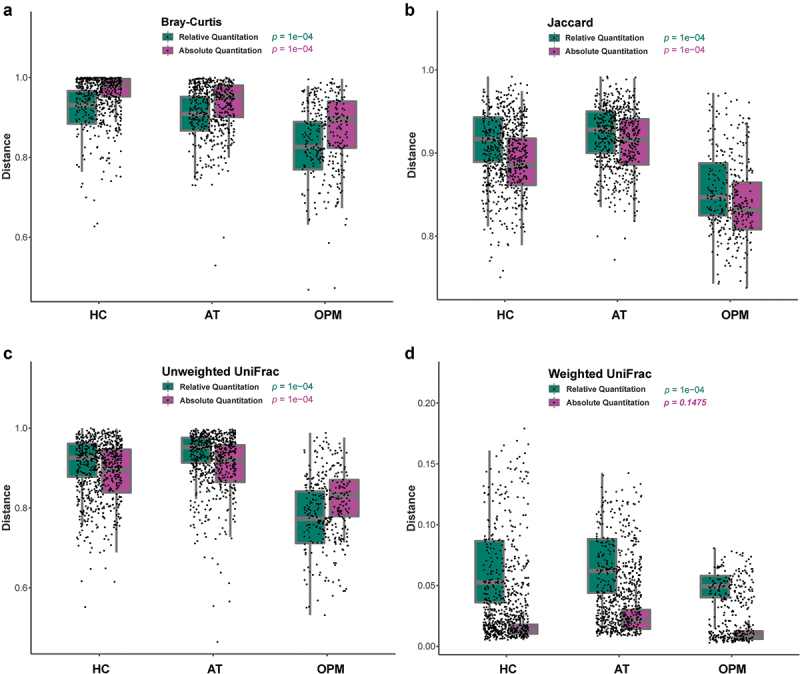
**The beta-diversity of HC, AT, and OPM groups analyzed using different methods**. (A) Bray-Curtis method. (B) Jaccard method. (C) Unweighted UniFrac. The variation in these three groups showed the same tendency in both QMP and RMP. (D) In Weighted Unifrac, QMP showed no difference in microbiota variation in these three groups (*p* = 0.1475).

**Figure 3.**
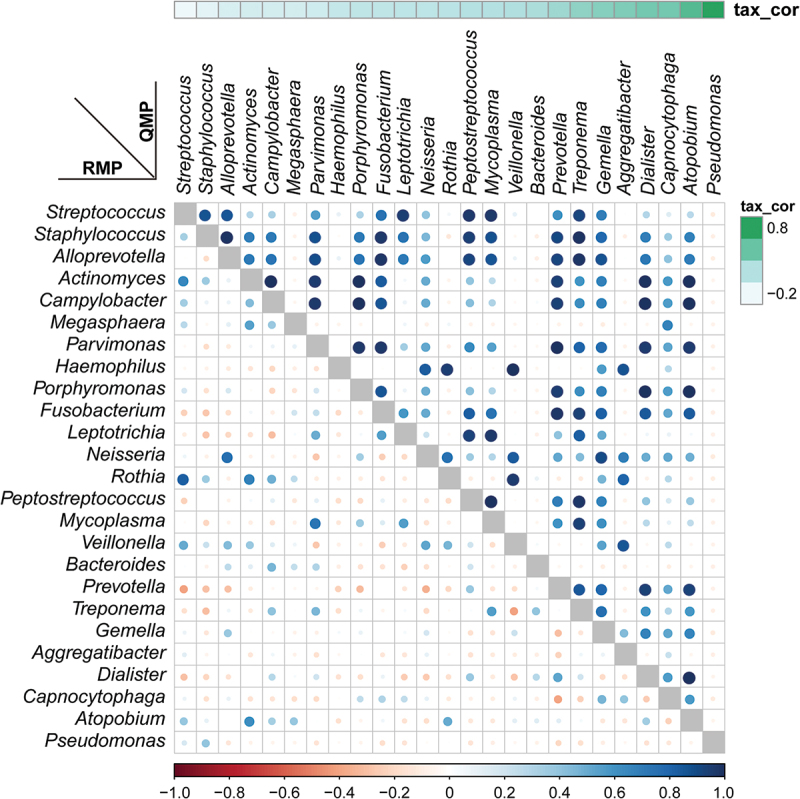
**Relative versus absolute abundance of microbiota network reconstruction**. A total of 25 common microbiomes in HC group were cross-validated using QMP (upper triangle) and RMP (lower triangle). The taxa are ordered by the significance of the correlation between their QMP abundance and DNA count. The correlation coefficient was measured by Spearman’s ρ analysis and presented using different colors and sizes of circles. The color gradients on the matrix axes (blue: positive correlation; red: negative correlation) are shown.

The interpretation of alpha-diversity in QMP composition was slightly different from the RMP analysis. There was a significant difference in richness of microbial communities found within RMP groups, yet there was no difference between groups in QMP analysis (**Supplementary Figure 2A-C**). In beta-diversity analysis, both RMP and QMP showed the same tendency that the microbiota abundance in OPM was the lowest when using Bray-Curtis, Jaccard, and Unweighted UniFrac method (*p* < 0.001, Figure 2A-C). However, the Weighted UniFrac method in QMP indicated no obvious microbial variation between groups (*p* = 0.1475, (Figure 2D). A total of 25 common genera with low to high relevant taxon in the HC group were picked up and reconstructed to establish correlation through using RMP and QMP data matrices. A far larger number of significantly co-varying genus pairs were detected in the QMP network (Figure 3).

### Analyzing the bacterial composition between HC and AT

**Figure 4.**
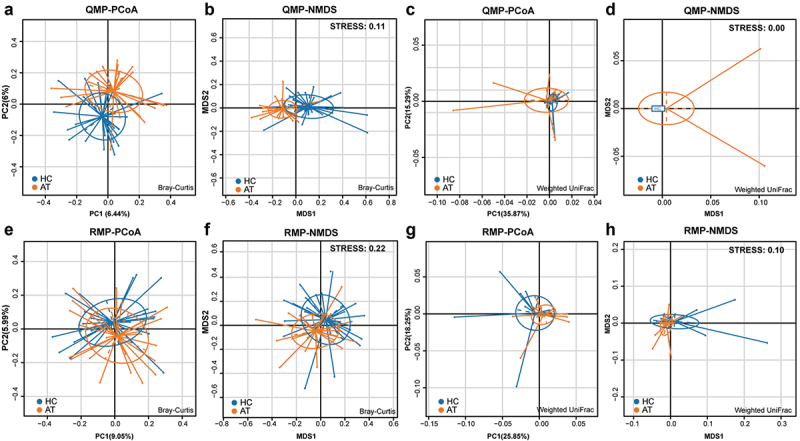
**Bray-Curtis and Weighted UniFrac based beta-diversity in HC and AT groups**. (A-B) Bray-Curtis based PCoA and NMDS presented significant differences in QMP (Adonis: *p* = 0.0005). However, the absolute abundance of Weighted UniFrac based PCoA and NMDS (C-D) and relative abundance of both Bray-Curtis and Weighted UniFrac based beta-diversity (E-H) showed no significant difference (Adonis: *p* > 0.05).

**Figure 5.**
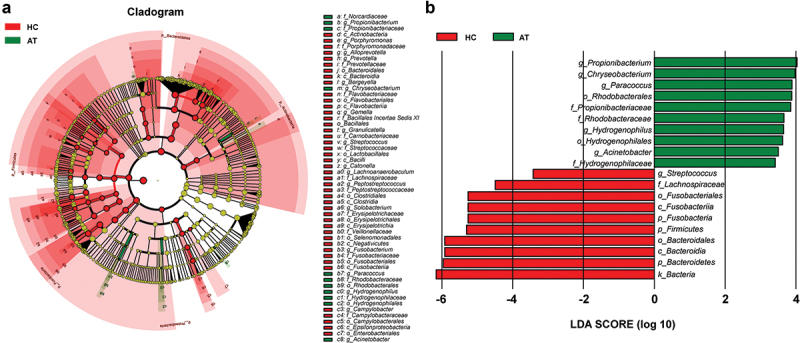
**The absolute abundance of microbiota through LefSe analysis in HC and AT groups**. (A) A cladogram represents the microbiota in HC and AT. The brightness of each dot was proportional to its effect size. (B) Taxa were enriched in HC group (Red), and AT groups (Green), indicating the variation of microbial communities under LDA scores (LDA = 2), respectively.

The composition of microbiota on the tissues of HC and AT was confusing in terms of whether a variation of microbiota was present in these two groups. The comparison analyses of QMP and RMP-based richness and diversities of microbiota in alpha-diversity were evaluated by using observed species, ACE index, and Shannon index. RMP indicated that the observed species, ACE index, and Shannon index were downregulated in the HC group compared to AT group (*p* < 0.05, **Supplementary Figure 3A-C**). However, in QMP, only the Shannon index found that the diversity of microbiota decreased in HC (*p* < 0.05, **Supplementary Figure 3D-F**). In beta-diversity analysis, QMP based Bray-Curtis method indicated that there was a significant difference in microbiota between HC and AT groups (Adonis: *p* = 0.0005), (Figure 4A-B), whereas both QMP and RMP-based Weighted UniFrac methods presented no such difference between groups (Adonis: *p* > 0.05, Figure 4C-H). LEfSe analysis indicated that genus *Streptococcus*, order *Fusobacteriale,* and *Bacteroidales* were higher in the HC group, whereas genera *Acinetobacter, Paracoccus, Chryseobacterium*, and *Propionibacterium* were lower (Figure 5A-B).

### Comparing the bacterial composition between HC and OPM

**Figure 6.**
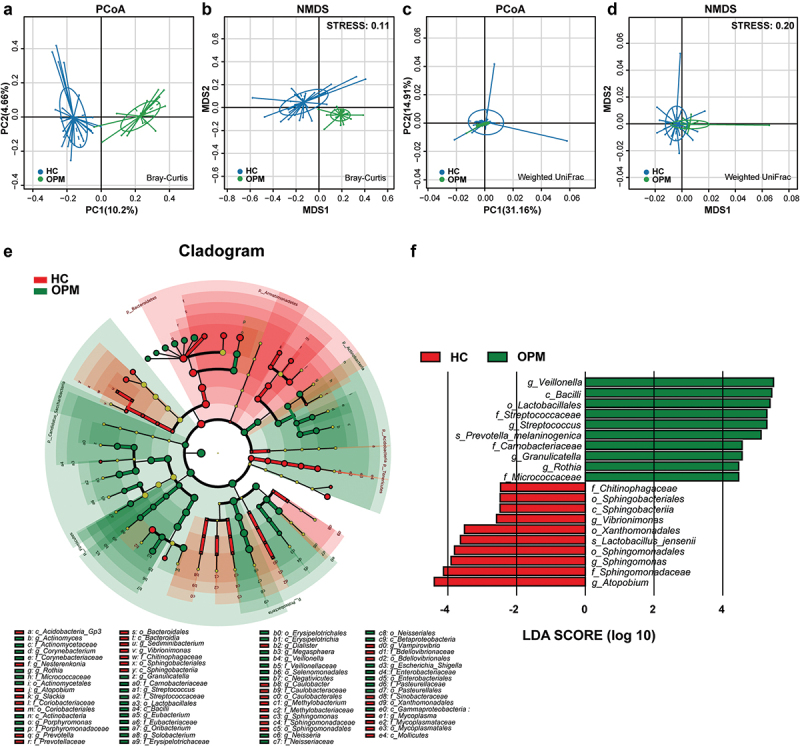
**The absolute beta diversity and LEfSe analysis in HC and OPM groups**. (A-B) Both PCoA and NMDS methods under Bray-Curtis analyses showed a significant difference in HC and OPM groups (Adonis: *p* = 0.0001). (C-D) Both PCoA and NMDS methods under Weighted UniFrac analyses showed significant differences in HC and OPM groups (Adonis: *p* = 0.0008). (E) A cladogram, on the left side, represents the OPM microbiota in HC and OPM. The brightness of each dot was proportional to its effect size. (F) Taxa were enriched in HC group (Red), and OPM groups (Green), indicating the variation of microbial communities under LDA scores (LDA = 2), respectively.

**Figure 7.**
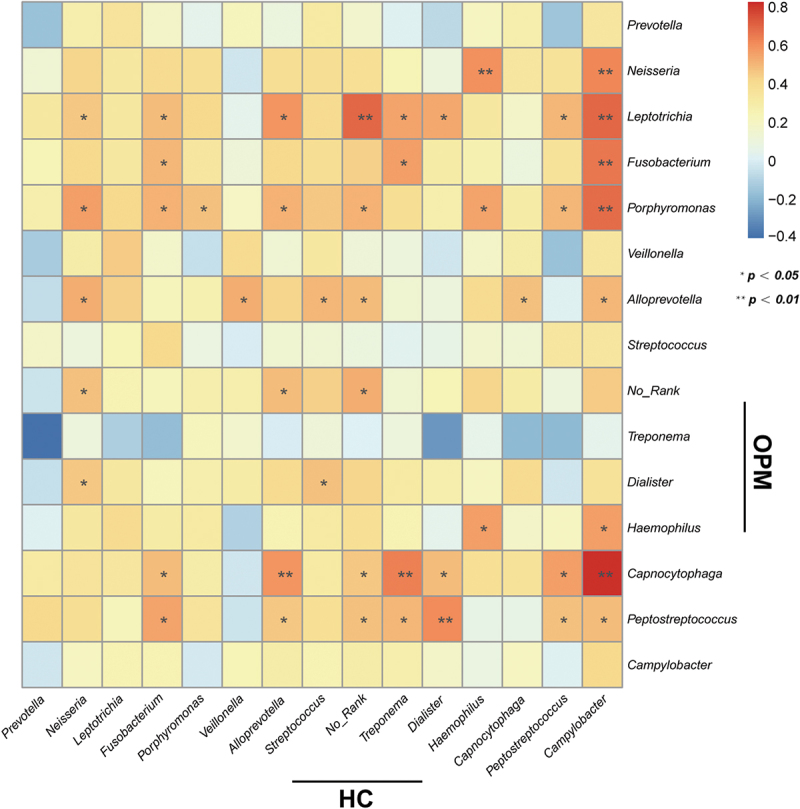
**Heatmap indicates the correlation of HC and OPM in QMP**. The top 15 of high absolute abundance of genera in HC were screened out and had their correlations compared with OPM through Spearman’s correlation analysis. * *p* < 0.05; ** *p* < 0.01.

We hypothesized that the microbiota of OPM is also the source of HC. Thus for better accessibility and to minimize invasiveness in future potential tests, we chose to compare HC microbiota to that collected by oral swabs. In RMP-based alpha-diversity analysis, we could not determine the variation of richness and evenness for HC and AT groups (*p* > 0.05, **Supplementary Figure 4A-C**). But in QMP, the richness in microbiota was found downregulated in HC when compared to AT (*p* < 0.05, **Supplementary Figure 4D-F**). Both Weighted UniFrac and Bray-Curtis based PCoA and NMDS demonstrated that HC had significantly higher diversity than the OPM group (Adonis: *p* < 0.001, Figure 6A-D). LEfSe analysis indicated that genera *Atopobium, Sphingomonas*,and *Vibrionimonas* were higher in the HC group, whereas high abundances of genera *Veillonella, Streptococcus, Granulicatella*, and *Rothia* were present in the OPM group (Figure 6E-F). Correlation analysis in the absolute abundance of genera between HC and OPM was then used to explore whether varied genera in OPM could influence the bacterial communities in carcinoma. A positive relationship between HC and OPM was found for genera *Fusobacterium, Porphyromonas, Haemophilus*, and *Peptostreptococcus* (Figure 7).

### Absolute abundance changes in microbial communities under stratified TNM staging

**Figure 8.**
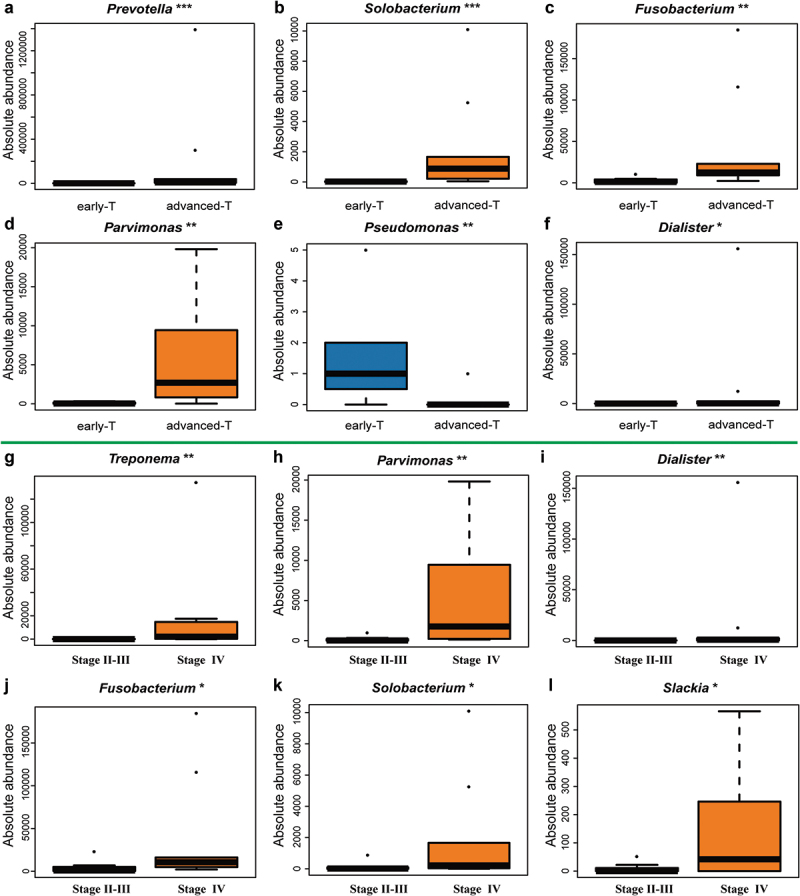
**Potential genuses of microbiota found in HC group under different T classification and TNM stagings**. (A-F) The genera *Prevotella* (*p* = 0.0001), *Solobacterium* (*p* = 0.0008), *Fusobacterium* (*p* = 0.0024), *Parvimonas* (*p* = 0.0024), *Dialister* (*p* = 0.016) increased whereas the genus *Pseudomonas* (*p* = 0.009) was downregulated in advanced T classification (T3-T4), when compared to early T classification (T1-T2). (G-L) The genera *Treponema* (*p* = 0.0043), *Parvimonas* (*p* = 0.0043), *Dialister* (*p* = 0.0085), *Fusobacterium* (*p* = 0.0155), *Solobacterium* (*p* = 0.0366), *Slackia* (*p* = 0.0412) were higher in advanced TNM staging (IV), when compared to early TNM staging (II–III).

Combined with the common clinical diagnostic system, we discovered that the absolute abundance of differential microbiota appeared to have connections with variation in biodiversity. Compared to early T classification (T1-2), the absolute abundance of *Prevotella, Solobacterium, Fusobacterium*, and *Parvimonas* at genus level increased in advanced T classification (T3-4), whereas *Pseudomonas* tended to down-regulate (Figure 8A-F). In TNM staging, *Treponema, Parvimonas, Dialister, Fusobacterium, Solobacterium*, and *Slackia* at genus level showed an increase in Stage IV compared to Stage II-III (Figure 8G-L). We also stratified the HC group under the N classification. Unfortunately, no differential microbiota was found between early N classification (N0) and advanced N classification (N1-N3).

### Functional prediction analysis using PICRUSt2

The PICRUSt2 approach was employed to provide the potential function of enrolled samples. The chosen references were used to match KEGG to offer potential biological processes. Compared with AT groups, both OPM and HC groups showed that microbiota could participate in some pathways, such as metabolism processes, DNA replication and damage, and biosynthesis of multiple amino acids to boost the progression of carcinoma (**Supplementary Figure 5**)

## Discussion

For the past dacade, human papilloma virus (HPV) infection associated HNC has been increasingly mentioned, especially in oropharyngeal carcinoma [20] and HPSCC [21]. Compared to patients with negative HPV, patients with positive HPV had a higher survival rate, suggesting that microbiota could be a biomarker in predicting prognosis in carcinoma. Bacteriome, as one kind of microbiome, is the largest source of genome in human bodies, thus different bacterial interactions may have impactful ramifications for the human microenvironment system. Through oral cavities that contain an estimated 700 species and 150 genera [22], it is possible to gain a glimpse of the microbial communities’ construct within the body, making it a highly accessible approach to discovering biomarkers for HPSCC. The swab is the most easy and convenient method to sample oral bacteriome, which prompted us to first confirm whether it is reliable in reflecting bacteriome involved in HPSCC by comparing the microbial communities between OPM and HC. Thereafter, we adopted spike-in QMP and RMP to cross-validate the composition in different groups. The comprehensiveness of QMP and RMP were also analyzed to help guide future research on this topic.

The next-generation high throughput amplicon sequencing is a well-established approach to profile the overall microbiota in many microbiome surveys. However, their relative abundance has increasingly raised doubts on whether taxon density could be accurately reflected. QMP data possess advantages that can be easily applied in any meta-omics workflow, cross-comparing key microbiome findings founded on relative profiling. To our knowledge, although spike-in based QMP had been applied in some carcinoma research [23], it is mostly used in environmental and ecological studies [24,25]. This internal standard normalization (ISN) approach could form an internal standard curve by comparing spike-ins with known concentrations of DNAs or cells. In our study, we adopted nine different synthetic spike-ins with known concentrations of ASVs to ensure data precision. The advantage of ISN is that it could predict a higher abundance of bacteria instead of failing to identify cell counts with particle-bacteria associations, such as flow cytometry. Furthermore, there is no need for ISN to duplicate or triplicate the samples to lower the higher range of typical coefficients of variations as qPCR [11]. Therefore, ISN-based QMP could alleviate time- and labor-costing work. Given the precision of ISN could be influenced by the DNA extraction efficiency and cell density in a prior study [26], our measurements attempted to minimize such an effect by using QIIME2.0 to recognize amplicon sequence variant (ASVs), DADA2 to denoise the data, thereby achieving reliability, and using DNA (ng) as the denominator instead of cell density.

OPM group contains the highest amount of microbiome out of the three groups, which was consistent with the hypothesis that microbial communities on carcinoma might originate from the oral cavity. However, in QMP-based alpha diversity, Ace index and Shannon index revealed that microbiota in individual OPM groups presented no significant difference when compared to HC and AT groups. Beta-diversity yielded through Bray-Curtis, Jaccard, and Unweighted UniFrac analyses indicated that OPM had the smallest community out of the three groups. When referring to absolute quantitation, HC, AT, and OPM were similar in terms of concentration and phylogenetic tree in Weighted UniFrac. This suggests that HC, AT, and OPM are cogenetic.

The variation in oropharyngeal microbiota might influence the microenvironment of the following anatomical structure. The bacterial composition was found similar in HC and AT containing mainly periodontal pathogens of *Prevotella* and *Fusobacterium*. The genus *Prevotella*, a kind of small gram-negative anaerobic rod, dominated in the oropharynx and hypopharynx cavities. *Prevotella* which is associated with oral and laryngeal squamous carcinoma [14,27], along with the genus *Fusobacterium*, has been well studied in the context of CRC progression [28]. Furthermore, we revalidated the HC group and its correlation with different taxonomies via QMP or RMP and discovered that traditionally known opposite combinations of microbial communities in RMP might demonstrate divergent interpretation in QMP. The RMP finding was in line with previous reports that found periodontal *Fusobacterium, Prevotella*, and *Alloprevotella* were enriched in carcinoma while *Streptococcus* depleted in OSCC [29]. Gong et al. also confirmed that the relative abundance of *Firmicutes* had an inverse correlation with other bacterial phylum communities (*Fusobacteria, Actinobacteria, Proteobacteria*, and *Bacteroidetes*) through oral sampling in laryngeal carcinoma [30]. Here in HPSCC, we found that the QMP presented results that are different from RMP. The genus *Streptococcus* showed a positive correlation with *Fusobacterium*. This finding runs contrary to previous reports, and the mechanisms need to be further elaborated.

Consistent with a prior study on similar microbiome differences in HNC tumor and paired-normal tissue, our study revalidated the result [31]. AT group did have higher microbiota diversities, which HC did not in alpha-diversity. Intriguingly, two beta-diversities analyses (Bray-Curtis and Weighted UniFrac) yielded separate results. In the Bray-Curtis method, the microbial communities were significantly different, yet there was no difference found when using Weighted UniFrac analysis in QMP. We noticed that such similar proximity levels of taxonomy in AT and HC were because they are neighbored and contain similar biofilm. The Bray-Curtis analysis was used to discriminate the only variation of community in different groups, but Weighted UniFrac has also emphasized the taxonomy similarity in microbial communities. Given the interpretation of Bray-Curtis might be more appropriate in this scenario, their similarity could not be neglected. These subtle differences could only be observed in abundance-based QMP, and are absent in RMP. Combining QMP with RMP, we speculate that the compositions in AT group and HC group were the same, and the difference between them comes from their absolute abundance.

Although swab as a sampling method was recommended by HMP, no studies have clarified that microbiota in OPM could be a representative method for analyzing microbiota in the carcinoma area. We speculated that if the microbiota of HC originated from the OPM group, then a correlation should be discovered. Therefore, a heatmap metric was built to understand the linear correlation regarding the one-to-one absolute abundance of genera in HC and OPM. Most common bacterial communities showed a positive correlation between the oropharynx and solid tumors. The genera *Fusobacterium, Porphyromonas, Haemophilus*, and *Peptostreptococcus* were positive but showed significant differences. The findings were also similar to the progression of OSCC [32] and pancreatic head carcinoma [33].

Unlike studies with relatively stable environments, such as soil or marine biology, the human body is organic and fluctuates. It is known that the composition of microorganisms could be influenced by ones’ living pattern, diet, habits, and dental health. In HPSCC, smoking and alcohol addiction were noticed as common risk factors. We found similar phenomenons for those who have drinking and smoking habits under QMP analysis. The drinking habit was found to be highly connected with the abundance of microbiota in alpha-diversity, which is consistent with a previous study that alcohol could be seen as a more important risk factor among oropharynx and hypopharynx carcinoma than the oral cavity or larynx (**Supplementary Figure 6A-D**) [34]. After subsequent categorization of the HC group, we found that genera *Fusobacterium* and *Parvimonas* were presented in greater quantities in advanced-stage T classification (T3-T4) and the highest advanced stage (TNM IV), when compared to the early-stage T classification (T1-2) and TNM II–III, respectively, which was consistent with the result of swab testing in OPM of HPSCC patient [35]. Both *Parvimonas* and *Fusobacterium* were found to be associated with periodontal disease [36], and diagnostic biomarkers for CRC [37] and OSCC [38]. The synergistic biofilm formation in these two bacteria might be the causative factor for these diseases [39]. *Fusobacterium nucleatum (F. nucleatum*), one of *Fusobacterium spp*, was identified to be associated with risks of HNC [40], and has the potential to be a risk factor for HPSCC. *F. nucleatum* facilitates the initiation and progression of CRC through metabolite, virulence factors (FadA, Fap2), miRNA and anti-immune effects, which might proceed to be used as a reference in mechanism findings for HPSCC [41].

The possible functions associated with carcinogenesis, predicted by the KEGG pathway of PICRUSt2, focused on mismatch repair, and the metabolism of amino acids, pyrimidine, and purine. A defect in the DNA repair system through a deficient mismatch repair system (dMMR) leads to microsatellite instability (MSI) which could be noticed as a gene-diagnostic method in CRC [42] and OSCC [43]. Metabolic reprogramming, in which metabolic heterogeneity induces metabolic symbiosis in carcinoma, has been known to facilitate cell survival, epithelial–mesenchymal transition (EMT), reshape the immune status, to adapt to the hostile microenvironment [44].

The limitations that existed in this single-center pilot report were that the sample is relatively small. Although HC was fully collected, a few samples were missing for OPM and AT, which might influence the results to some extent. Furthermore, the study focused on the cross-sectional comparison, which, unlike longitudinal studies, did not discuss the outcome of treatment such as induction chemotherapy or concurrent chemoradiotherapy, thus weakening the link toward clinical applications. Although our data should be revalidated in a large-scale study, this study contiributes to highlighting the use of the spike-in QMP method in HNC, minimizing the extra labor and time expenditure. Higher reliability and comprehensive applications should be expected in future microbiome–carcinoma studies following the use of QMP.

## Conclusion

The dysbiosis of the OPM group is characterized by a lower diversity of microbiota when compared to HC and AT groups. QMP complements traditional RMP where results contrary to previous RMP studies were discovered, demonstrating advantages in comprehensiveness. Categorized HC group with TNM staging under QMP analysis, we discovered that *Fusobacterium, Parvimonas,* and *Dialister* at genus level tend to present in larger quantities in advanced T classification, and TNM staging. Oropharyngeal swab could be a serviceable non-invasive alternative to biopsy for microbiota assessment in HPSCC.

## Supplementary Material

Supplemental Material

## Data Availability

The accession number of Genome Sequence Archive (GSA) in China National Center for Bioinformation (CNCB) is **CRA004979 (https://ngdc.cncb.ac.cn/gsa/).**
