# Supplementary material for: Cross-comparison of microbiota in the oropharynx, hypopharyngeal squamous cell carcinoma and their adjacent tissues through quantitative microbiome profiling
Source: J Oral Microbiol. 2022 May 10;14(1):2073860. doi: 10.1080/20002297.2022.2073860 (PMC9103590; doi:10.1080/20002297.2022.2073860)
Supplement: Supplemental Material [file ZJOM_A_2073860_SM9539.zip › Supplementary files/Supplementary Figure 3.pdf]

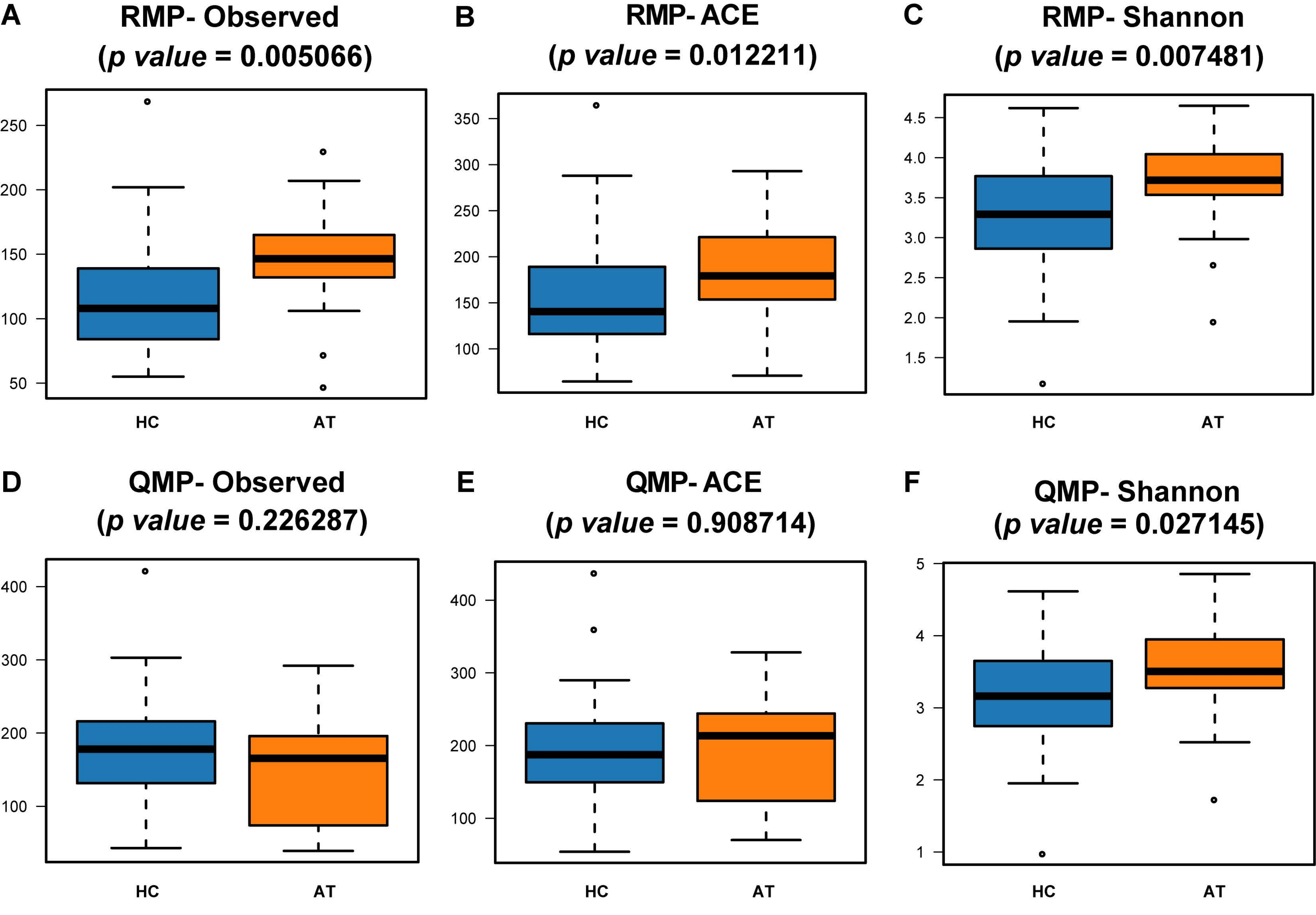
A
RMP- Observed
(*p value* = 0.005066)
250
200
150
100
50
HC
AT
B
RMP- ACE
(*p value* = 0.012211)
350
300
250
200
150
100
HC
AT
C
RMP- Shannon
(*p value* = 0.007481)
4.5
4.0
3.5
3.0
2.5
2.0
1.5
HC
AT
D
QMP- Observed
(*p value* = 0.226287)
400
300
200
100
HC
AT
E
QMP- ACE
(*p value* = 0.908714)
400
300
200
100
HC
AT
F
QMP- Shannon
(*p value* = 0.027145)
5
4
3
2
1
HC
AT
