# Supplementary material for: Cross-comparison of microbiota in the oropharynx, hypopharyngeal squamous cell carcinoma and their adjacent tissues through quantitative microbiome profiling
Source: J Oral Microbiol. 2022 May 10;14(1):2073860. doi: 10.1080/20002297.2022.2073860 (PMC9103590; doi:10.1080/20002297.2022.2073860)
Supplement: Supplemental Material [file ZJOM_A_2073860_SM9539.zip › Supplementary files/Supplementary Table 1.pdf]

Supplementary Table 1. The locations from each patient that samples were obtained

Patient number	HC	AT	OPM
18	Y	Y	Y
19	Y	Y	N
21	Y	Y	Y
22	Y	Y	Y
23	Y	Y	Y
25	Y	Y	Y
26	Y	Y	Y
27	Y	Y	Y
31	Y	Y	Y
33	Y	Y	N
34	Y	Y	N
36	Y	Y	Y
37	Y	Y	N
38	Y	Y	Y
41	Y	Y	N
43	Y	Y	N
47	Y	Y	N
49	Y	Y	Y
50	Y	Y	N
51	Y	N	Y
55	Y	Y	Y
56	Y	Y	Y
57	Y	Y	Y
58	Y	Y	Y
59	Y	Y	Y
62	Y	Y	Y
70	Y	Y	Y

Abbreviation: Y for sample collected; N for failure to obtain sample.
