# Supplementary figures and images for: Cross-comparison of microbiota in the oropharynx, hypopharyngeal squamous cell carcinoma and their adjacent tissues through quantitative microbiome profiling
Source: J Oral Microbiol. 2022 May 10;14(1):2073860. doi: 10.1080/20002297.2022.2073860 (PMC9103590; doi:10.1080/20002297.2022.2073860)

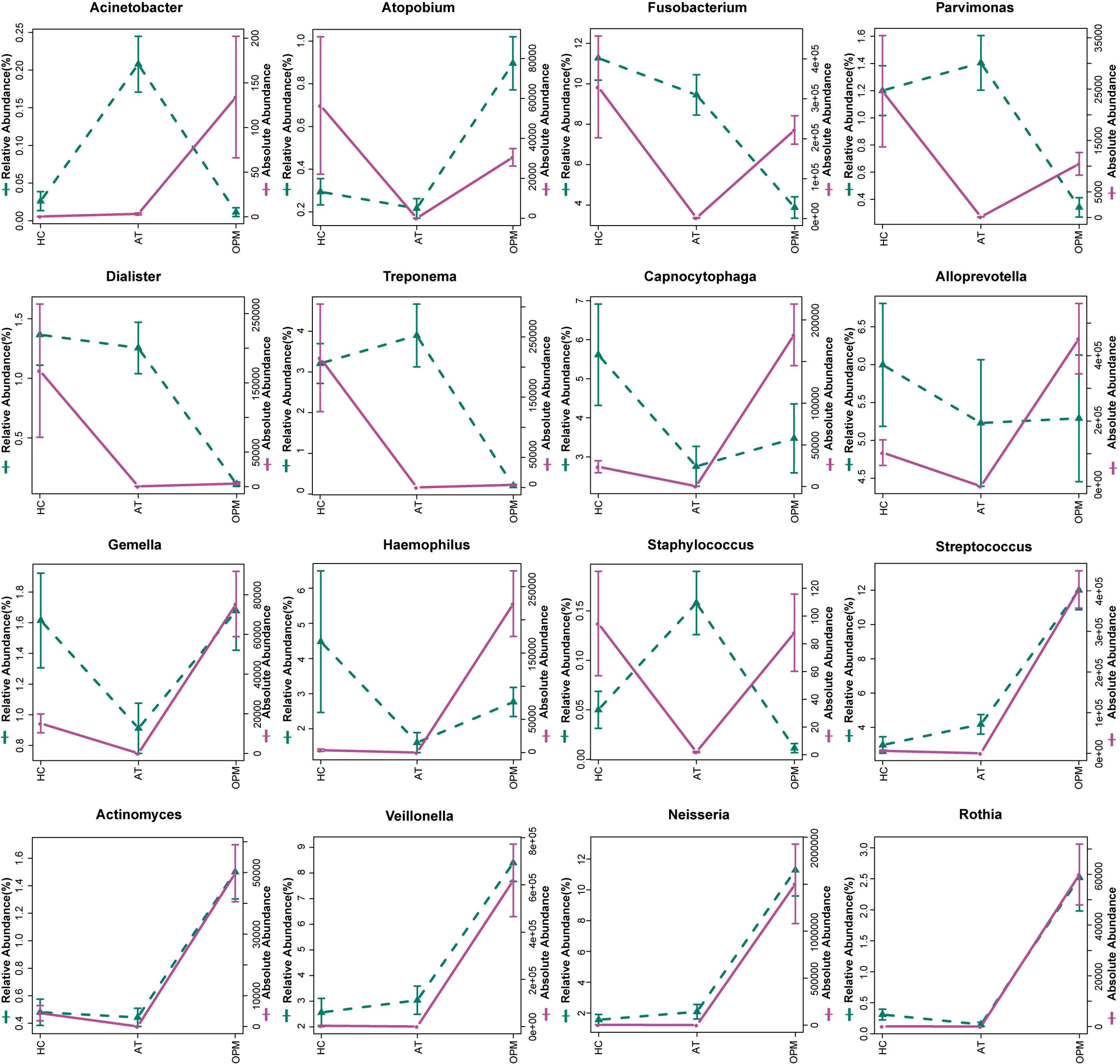

Supplement: Supplemental Material [file ZJOM_A_2073860_SM9539.zip › Supplementary files/Supplementary figure 1.pdf]

**A**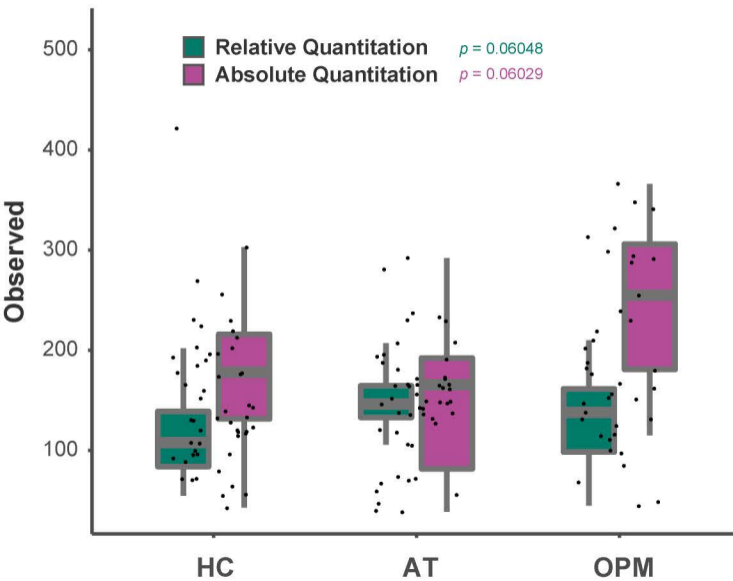**B**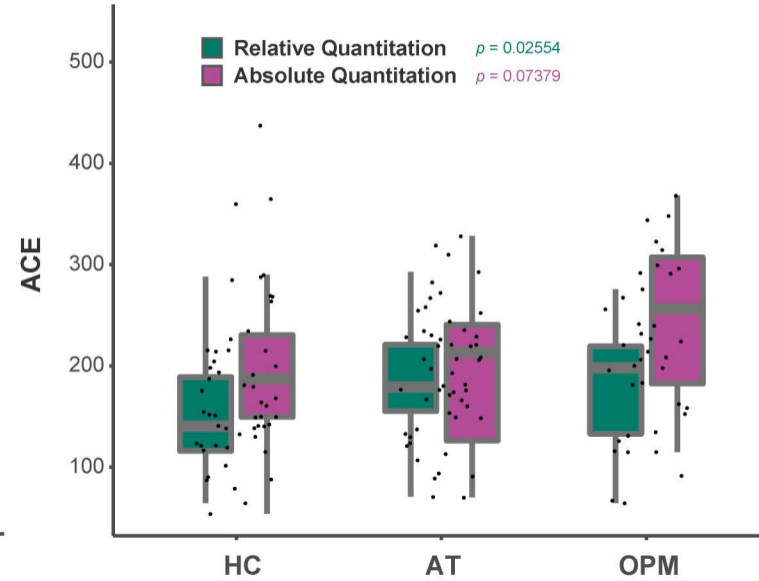**C**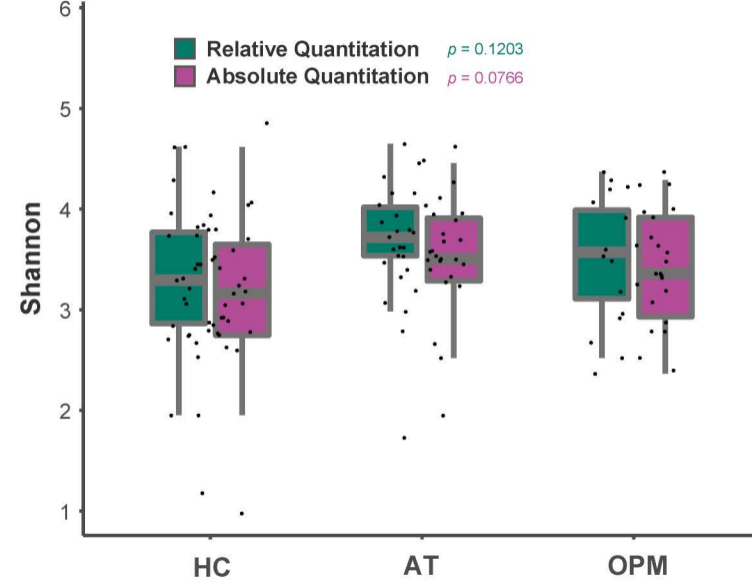

Supplement: Supplemental Material [file ZJOM_A_2073860_SM9539.zip › Supplementary files/Supplementary Figure 2.pdf]

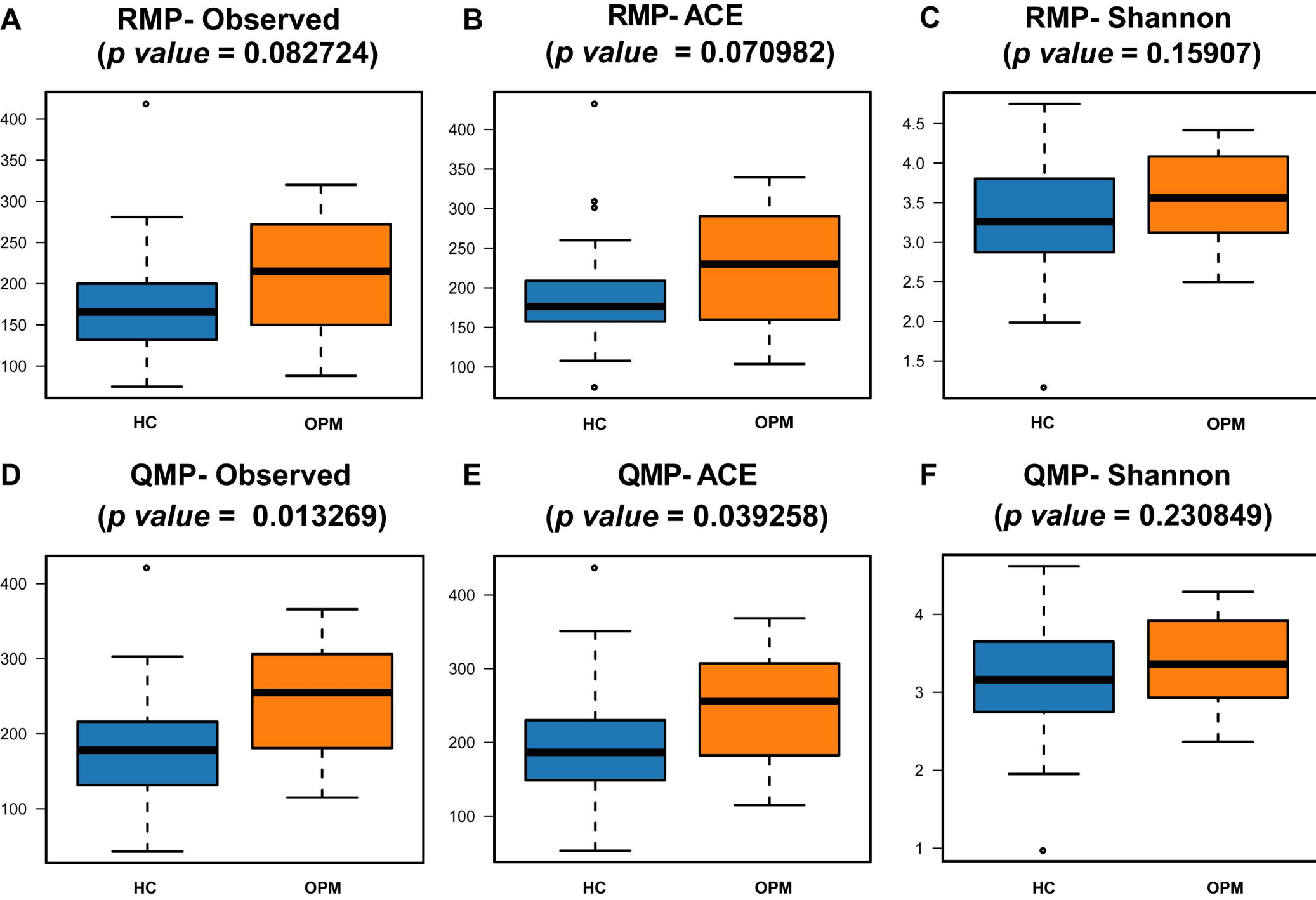

Supplement: Supplemental Material [file ZJOM_A_2073860_SM9539.zip › Supplementary files/Supplementary Figure 4.pdf]

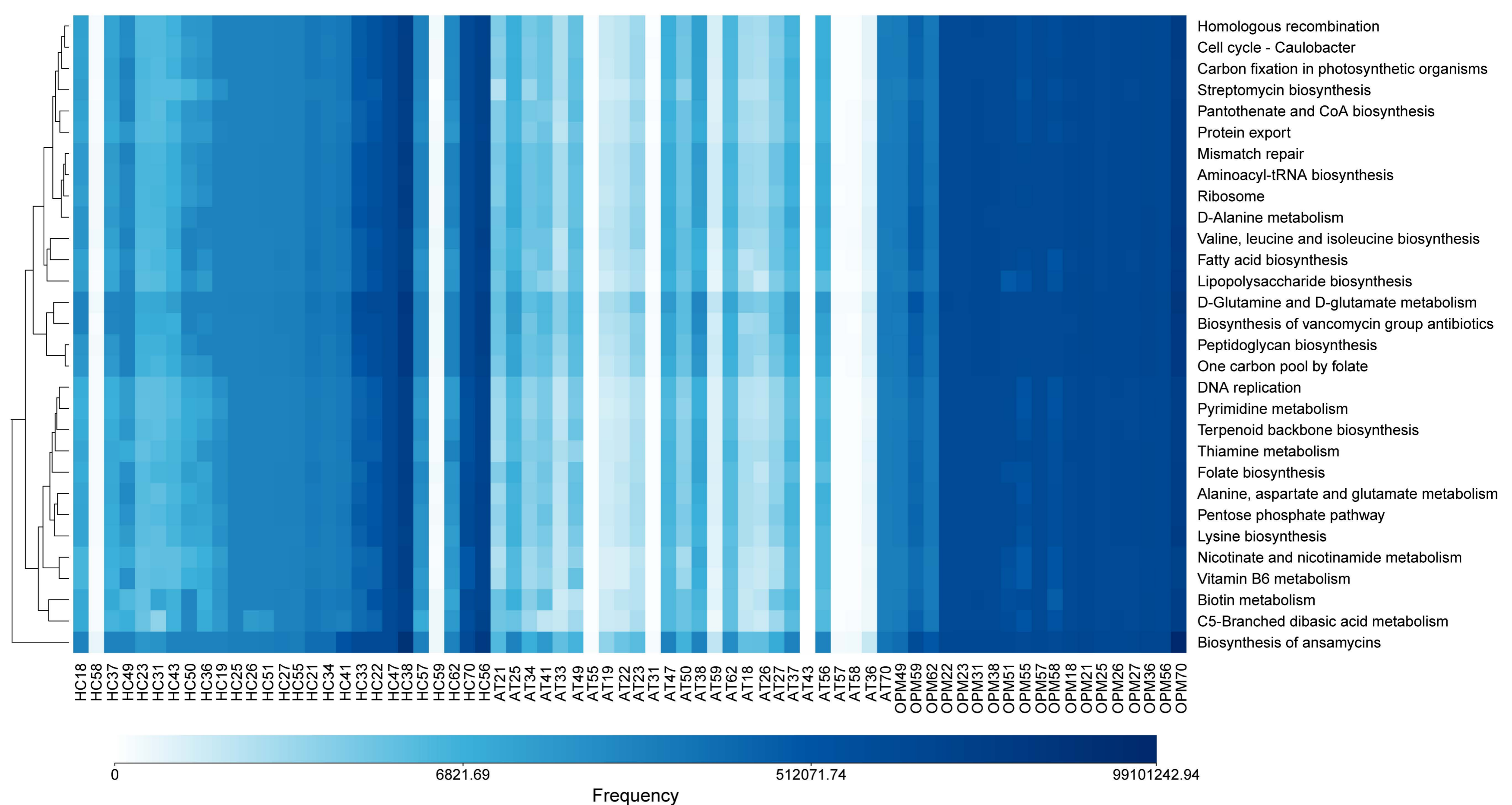

Supplement: Supplemental Material [file ZJOM_A_2073860_SM9539.zip › Supplementary files/Supplementary Figure 5.pdf]

**A**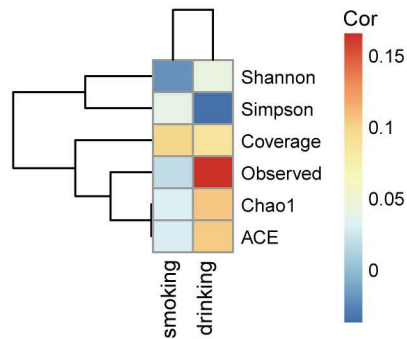**B**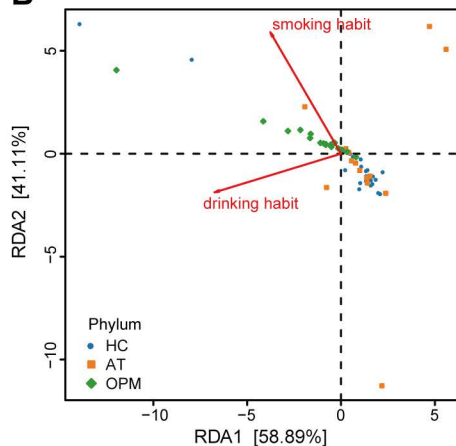**C**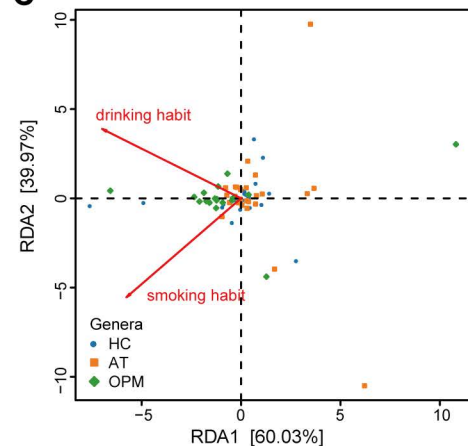**D**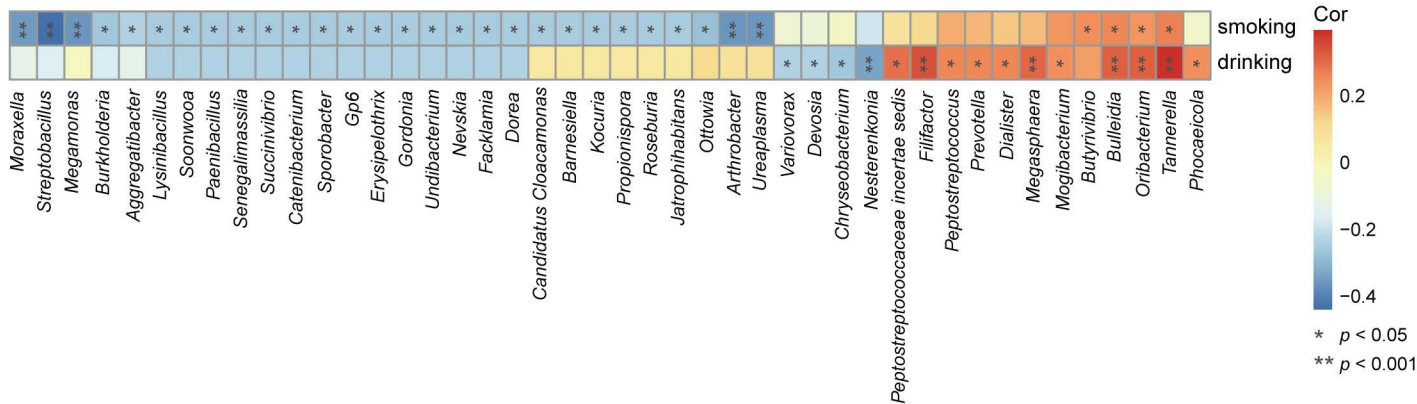

Supplement: Supplemental Material [file ZJOM_A_2073860_SM9539.zip › Supplementary files/Supplementary Figure 6.pdf]
